# Supplementary material for: Cross-sectional study of the association of social relationship resources with Staphylococcus aureus colonization in naturally occurring social groups along the US/Mexico border
Source: PLoS One. 2023 Apr 13;18(4):e0284400. doi: 10.1371/journal.pone.0284400 (PMC10101449; doi:10.1371/journal.pone.0284400)
Supplement: Attachment — Submitted filename: PONE-D-23-00421.docx [file pone.0284400.s002.docx]

**PLOS ONE**

**Cross-sectional study of the association of social relationship resources with Staphylococcus aureus colonization in naturally occurring social groups**

--Manuscript Draft--

| **Manuscript Number:** | PONE-D-23-00421 |
| --- | --- |
| **Article Type:** | Research Article |
| **Full Title:** | Cross-sectional study of the association of social relationship resources with Staphylococcus aureus colonization in naturally occurring social groups |
| **Short Title:** | Social relationships and S. aureus colonization |
| **Corresponding Author:** | Steven Barger  Northern Arizona University Flagstaff, AZ UNITED STATES |
| **Keywords:** | Social relationships; social integration; social networks; loneliness; Staphylococcus aureus; Hispanic Americans |
| **Abstract:** | Asymptomatic carriage of Staphylococcus aureus is a major risk factor for subsequent clinical infection. Diminishing returns from mitigation efforts emphasize the need to better understand colonization, spread, and transmission of this opportunistic pathogen. While contact with other people presents opportunities for pathogen exposure and transmission, diversity of social connections may be protective against pathogens such as the common cold. Here we examine whether social relationship resources, including the amount and diversity of social contacts, are associated with S. aureus colonization. We enrolled community members (N=443; 68% Hispanic) in naturally occurring social groups in southwestern Arizona. We assessed four types of social relationships and loneliness, and sampled the skin, nose and throat to  ascertain S. aureus colonization. Overall S. aureus prevalence was 64.8%. Neither the amount nor the diversity of social contacts were associated with S. aureus colonization. The concurrent validity of the social relationship assessments was supported by their moderate intercorrelations and by their positive association with self-rated health. Our results suggest that the association of social network diversity and susceptibility to the common cold does not extend to S. aureus colonization. Conversely, we did not detect increased colonization prevalence among those with more social contacts. The latter pattern suggests that social transmission may be relatively infrequent or that more intimate forms of social interaction may drive transmission and colonization resulting in high community prevalence of S. aureus colonization. These data inform communicable disease control efforts. |
| **Order of Authors:** | Steven D Barger |
|  | Monica R Lininger |
|  | Robert T Trotter III |
|  | Mimi Mbegbu |
|  | Shari Kyman |
|  | Kara Tucker-Morgan |
|  | Colin Wood |
|  | Briana Coyne |
|  | Benjamin Russakoff |
|  | Kathya Ceniceros |
|  | Cristina Padilla |
|  | Sara Maltinsky |
|  | Talima Pearson |
| **Additional Information:** |  |

*Powered by Editorial Manager® and ProduXion Manager® from Aries Systems Corporation*

| **Question** | **Response** |
| --- | --- |
| **Financial Disclosure**  Enter a financial disclosure statement that describes the sources of funding for the work included in this submission. Review the [submission guidelines](http://journals.plos.org/plosone/s/submission-guidelines#loc-financial-disclosure-statement) for detailed requirements. View published research articles from [*PLOS ONE*](http://journals.plos.org/plosone/) for specific examples.  This statement is required for submission and **will appear in the published article** if the submission is accepted. Please make sure it is accurate. | This work was supported by the NAU Southwest Health Equity Research Collaborative, which is funded by the National Institute on Minority Health and Health Disparities at the National Institutes of Health (grant NIH U54MD012388). Funding was also received from the National Institute of Allergy and Infectious Diseases at the National Institutes of Health (R15AI156771) The funders had no role in study design, data collection and analysis, decision to publish, or preparation of the manuscript. |
| **Unfunded studies**  Enter: *The author(s) received no specific funding for this work.*  **Funded studies**  Enter a statement with the following details:   - Initials of the authors who received each award - Grant numbers awarded to each author - The full name of each funder - URL of each funder website - Did the sponsors or funders play any role in the study design, data collection and analysis, decision to publish, or preparation of the manuscript? - **NO** - Include this sentence at the end of your statement: *The funders had no role in study design, data collection and analysis, decision to publish, or preparation of the manuscript.* - **YES** - Specify the role(s) played. |  |
| * typeset |  |
| **Competing Interests**  Use the instructions below to enter a competing interest statement for this submission. On behalf of all authors, disclose any [competing interests](http://journals.plos.org/plosone/s/competing-interests) that could be perceived to bias this work—acknowledging all financial support and any other relevant financial or non- financial competing interests. | The authors have declared that no competing interests exist. |

*Powered by Editorial Manager® and ProduXion Manager® from Aries Systems Corporation*

| This statement is required for submission and **will appear in the published article** if the submission is accepted. Please make sure it is accurate and that any funding sources listed in your Funding Information later in the submission form are also declared in your Financial Disclosure statement.  View published research articles from  [*PLOS ONE*](http://journals.plos.org/plosone/) for specific examples. |  |
| --- | --- |
| **NO authors have competing interests**  Enter: *The authors have declared that no competing interests exist.*  **Authors with competing interests**  Enter competing interest details beginning with this statement:  *I have read the journal's policy and the authors of this manuscript have the following competing interests: [insert competing interests here]* |  |
| * typeset |  |
| **Ethics Statement**  Enter an ethics statement for this submission. This statement is required if the study involved:   - Human participants - Human specimens or tissue - Vertebrate animals or cephalopods - Vertebrate embryos or tissues - Field research   Write "N/A" if the submission does not require an ethics statement.  General guidance is provided below. Consult the [submission guidelines](http://journals.plos.org/plosone/s/submission-guidelines#loc-guidelines-for-specific-study-types) for detailed instructions. **Make sure that all information entered here is included in the Methods section of the manuscript.** | All adult participants provided informed consent and the study was approved by the Northern Arizona University Institutional Review Board. |

*Powered by Editorial Manager® and ProduXion Manager® from Aries Systems Corporation*

| **Format for specific study types**  **Human Subject Research (involving human participants and/or tissue)**   - Give the name of the institutional review board or ethics committee that approved the study - Include the approval number and/or a statement indicating approval of this research - Indicate the form of consent obtained (written/oral) or the reason that consent was not obtained (e.g. the data were analyzed anonymously)   **Animal Research (involving vertebrate animals, embryos or tissues)**   - Provide the name of the Institutional Animal Care and Use Committee (IACUC) or other relevant ethics board that reviewed the study protocol, and indicate whether they approved this research or granted a formal waiver of ethical approval - Include an approval number if one was obtained - If the study involved *non-human primates*, add *additional details* about animal welfare and steps taken to ameliorate suffering - If anesthesia, euthanasia, or any kind of animal sacrifice is part of the study, include briefly which substances and/or methods were applied   **Field Research**  Include the following details if this study involves the collection of plant, animal, or other materials from a natural setting:   - Field permit number - Name of the institution or relevant body that granted permission |  |
| --- | --- |
|  |  |
| **Data Availability**  Authors are required to make all data underlying the findings described fully available, without restriction, and from the time of publication. PLOS allows rare exceptions to address legal and ethical concerns. See the [PLOS Data Policy](http://journals.plos.org/plosone/s/data-availability) and [FAQ](http://journals.plos.org/plosone/s/data-availability#loc-faqs-for-data-policy) for detailed information. | Yes - all data are fully available without restriction |

*Powered by Editorial Manager® and ProduXion Manager® from Aries Systems Corporation*

| A Data Availability Statement describing where the data can be found is required at submission. Your answers to this question constitute the Data Availability Statement and **will be published in the article**, if accepted. |  |
| --- | --- |
| **Important:** Stating ‘data available on request from the author’ is not sufficient. If your data are only available upon request, select ‘No’ for the first question and explain your exceptional situation in the text box. |  |
| Do the authors confirm that all data underlying the findings described in their manuscript are fully available without restriction? |  |
| **Describe where the data may be found in full sentences. If you are copying our sample text, replace any instances of XXX with the appropriate details.**   - If the data are **held or will be held in a public repository**, include URLs, accession numbers or DOIs. If this information will only be available after acceptance, indicate this by ticking the box below. For example: *All XXX files are available from the XXX database (accession number(s) XXX, XXX.*). - If the data are all contained **within the manuscript and/or Supporting Information files**, enter the following: *All relevant data are within the manuscript and its Supporting Information files.* - If neither of these applies but you are able to provide **details of access elsewhere**, with or without limitations, please do so. For example:   *Data cannot be shared publicly because of [XXX]. Data are available from the XXX Institutional Data Access / Ethics Committee (contact via XXX) for researchers who meet the criteria for access to confidential data.*  *The data underlying the results presented in the study are available* | The data will be held in a public repository and the URL will be available after acceptance. |

*Powered by Editorial Manager® and ProduXion Manager® from Aries Systems Corporation*

| *from (include the name of the third party and contact information or URL).*   - This text is appropriate if the data are owned by a third party and authors do not have permission to share the data.   * typeset |  |
| --- | --- |
| Additional data availability information: | Tick here if the URLs/accession numbers/DOIs will be available only after acceptance of the manuscript for publication so that we can ensure their inclusion before publication. |

*Powered by Editorial Manager® and ProduXion Manager® from Aries Systems Corporation*

Manuscript	[Click here to](https://www.editorialmanager.com/pone/download.aspx?id=32447619&guid=5c59e0d4-38e4-4061-88d8-5a66b917fe75&scheme=1)

[access/download;Manuscript;PLOSOne_1_5_23v2.docx](https://www.editorialmanager.com/pone/download.aspx?id=32447619&guid=5c59e0d4-38e4-4061-88d8-5a66b917fe75&scheme=1)

1 Cross-sectional study of the association of social relationship resources with
2 *Staphylococcus aureus* colonization in naturally occurring social groups

3

4 Steven D. Barger^1^*, Monica R. Lininger^2^, Robert T. Trotter II^3,4^, Mimi Mbegbu^5^, Shari
5 Kyman^5^, Kara Tucker-Morgan^6^, Colin Wood^5^, Briana Coyne^5^, Benjamin Russakoff^5^,
6 Kathya Ceniceros^6^, Cristina Padilla^6^, Sara Maltinsky^5^, Talima Pearson^3,5^*

7

8	^1^Department of Psychological Sciences, Northern Arizona University, Flagstaff, AZ

9

10 ^2^Department of Physical Therapy and Athletic Training, Northern Arizona University,
11 Flagstaff, AZ

12

13	^3^Center for Health Equity Research, Northern Arizona University, Flagstaff, AZ

14

15	^4^Department of Anthropology, Northern Arizona University, Flagstaff, AZ

16

17	^5^Pathogen & Microbiome Institute, Northern Arizona University, Flagstaff, AZ

18

19	^6^Northern Arizona University, Yuma, Arizona

20

21

22 * Corresponding authors
23 E-mail: [talima.pearson@nau.edu](mailto:talima.pearson@nau.edu) (TP) or [steven.barger@nau.edu](mailto:steven.barger@nau.edu) (SDB)

24

# 25 Abstract

26 Asymptomatic carriage of *Staphylococcus aureus* is a major risk factor for subsequent
27 clinical infection. Diminishing returns from mitigation efforts emphasize the need to
28 better understand colonization, spread, and transmission of this opportunistic pathogen.
29 While contact with other people presents opportunities for pathogen exposure and
30 transmission, diversity of social connections may be protective against pathogens such
31 as the common cold. Here we examine whether social relationship resources, including
32 the amount and diversity of social contacts, are associated with *S. aureus* colonization.
33 We enrolled community members (N=443; 68% Hispanic) in naturally occurring social
34 groups in southwestern Arizona. We assessed four types of social relationships and
35 loneliness, and sampled the skin, nose and throat to ascertain *S. aureus* colonization.
36 Overall *S. aureus* prevalence was 64.8%. Neither the amount nor the diversity of social
37 contacts were associated with *S. aureus* colonization. The concurrent validity of the
38 social relationship assessments was supported by their moderate intercorrelations and
39 by their positive association with self-rated health. Our results suggest that the
40 association of social network diversity and susceptibility to the common cold does not
41 extend to *S. aureus* colonization. Conversely, we did not detect increased colonization
42 prevalence among those with more social contacts. The latter pattern suggests that
43 social transmission may be relatively infrequent or that more intimate forms of social
44 interaction may drive transmission and colonization resulting in high community
45 prevalence of *S. aureus* colonization. These data inform communicable disease control
46 efforts.

47

48

49 **Key words**: Social relationships; social integration; social networks; loneliness;
50 *Staphylococcus aureus*; Hispanic Americans

# 51 Introduction

52 Social network diversity, denoted by the number of different social relationship
53 roles in which one participates (i.e., spouse; parent; work; school; neighbor), presents
54 opportunities for pathogen exposure and subsequent transmission. Conversely, social
55 network diversity is associated with a lower probability of symptomatic infection after
56 intentional exposure to cold viruses (1). This suggests that social relationship resources
57 may be protective for some infectious diseases. However, human exposure to, and
58 illness from infectious agents, occurs in a complex ecological context that was
59 intentionally excluded from infectious challenge paradigms because all study
60 participants were exposed to the infectious agent (1, 2). Importantly, this putative
61 salutary link between social relationships and infectious diseases has only been
62 explored with cold viruses and is based upon unplanned analyses from a single study.
63 The potential importance of the association between social networks and communicable
64 diseases warrants further evaluation as it is critical for mitigation.
65 In the present work we address gaps in our understanding of the links between
66 social resources and infectious diseases by examining the association of social network
67 diversity with colonization by *Staphylococcus aureus* in naturally occurring groups of
68 community residents. *S. aureus* is a bacterium that many humans carry as a
69 commensal, without infections. However, if these bacteria penetrate the outer layers of
70 skin or mucosa, they act as a pathogen and can cause mild skin infections as well as
71 dangerous invasive infections (3). In 2017 *S. aureus* blood infections caused almost
72 20,000 deaths in the United States (U.S.) (4). It is estimated that about a third of the
73 U.S. population is colonized with *S. aureus* (5). However, our recent work using a
74 combination of culture-based and genomic assays shows a *S. aureus* prevalence of
75 ~66% and suggests that previous prevalence reports are underestimates (6). While
76 hospital-onset and healthcare-associated invasive infections of methicillin-resistant *S.*
77 *aureus* (MRSA) have decreased, community-associated MRSA and methicillin-
78 susceptible *S. aureus* (MSSA) infections have not (4, 7). The epidemiological
79 importance of such a community-based reservoir of *S. aureus* underscores the
80 importance of community sampling to understand prevalence, transmission, and drivers
81 of colonization*.* Colonization is a major risk factor for infection, with genetic studies
82 showing that colonizing strains of *S. aureus*, i.e., the strains found on skin, nares, etc.,
83 are commonly identical to infecting strains, suggesting that patients typically infect
84 themselves (8-10)*.* Thus, *S. aureus* colonization provides a marker for an infectious
85 disease that is both prevalent and closely tied to clinical infection. Importantly, when
86 assessed in community settings, *S. aureus* colonization remains situated in, and
87 therefore potentially influenced by, the social processes putatively underlying infectious
88 disease exposure.
89 We examined several social relationship indicators linked to health in general
90 and indicative of social interactions that may drive colonization. These included social
91 network diversity and social network size (the number of people in the social network).
92 Both network diversity and network size are considered measures of social integration,
93 reflecting individual participation in a broad range of social relationships (11)
94 (sometimes labeled social engagement (12)). We complemented these social network
95 metrics with a social integration measure that is used in public health surveillance and
96 only counts interaction with persons outside one’s household and disaggregates
97 different forms of social interaction (i.e., phone versus in-person contacts). Importantly,
98 this social integration measure robustly predicts hard health endpoints (i.e., mortality) in
99 large population-based samples and is clearly distinct from socioeconomic status
100 determinants of health (13, 14). Thus, this social integration measure complements the
101 other social network metrics and provides a bridge to existing population health
102 surveillance.
103 In addition to these structural social relationship measures we also assessed
104 loneliness. Loneliness reflects subjective satisfaction with both the quality and quantity
105 of one’s social relationships (15, 16). Loneliness can therefore disentangle the relative
106 importance of perceived social relationship deficits, which may or may not align with
107 being socially isolated as indicated by quasi-objective social integration scores (15, 17).
108 We included self-rated health as a convergent outcome for our analysis of social
109 relationships and *S. aureus* colonization. Self-rated health, typically measured by asking
110 respondents to rate their own health from *poor* to *excellent*, is a robust predictor of
111 mortality (18-21) and captures health status beyond established clinical and biomedical
112 assessments (21, 22). Because self-rated health is positively associated with several
113 measures of social integration (23, 24), including the public health version we use here
114 (25), we use self-rated health as an additional health status outcome to confirm the
115 convergent validity of the social relationship assessments.
116 Given the novelty of our outcome variable in relation to social relationship
117 resources, we did not make predictions regarding the association of social relationships
118 with *S. aureus* colonization. Instead, we specify several working hypotheses (26). First,
119 if social network diversity is inversely associated with *S. aureus* colonization, this would
120 extend the literature on salutary social relationships to an infectious agent other than
121 common cold viruses. To the extent that colonization is also inversely associated with
122 other social relationship resources, such as loneliness and/or network size, that would
123 show that the social determinants of infectious disease susceptibility are more extensive
124 than previously understood. If *S. aureus* colonization is unrelated to social network
125 diversity specifically, and to other social relationships generally (i.e., loneliness), this
126 would provide evidence for a boundary for salutary social relationship resources on
127 infectious disease susceptibility. A competing prediction is that social contacts *increase*
128 exposure to persons colonized with *S. aureus*. Therefore the number of contacts, as
129 indicated by social network size, would be positively associated with *S. aureus*
130 prevalence^1^. Similarly, if colonization is unrelated to more frequent social contacts, it
131 does not preclude the possibility that more intimate forms of social interaction (e.g.,
132 sexual relationships, cohabitation) may be more likely to result in transmission and
133 colonization. Irrespective of these patterns we expected social networks and social
134 integration to be positively associated with self-rated health (23-25).

# 135 Materials and Methods

## 136 Study overview

137 This study is part of a larger investigation of determinants of *S. aureus*
138 colonization and transmission in social groups recruited in south Yuma County in
139 Arizona (6, 27, 28). Briefly, research team members from the community were trained to
140 recruit participants, to supervise collection of survey data and to supervise participants’

1 This possibility is not directly testable in laboratory infectious challenge studies where all participants are exposed to the pathogen.

141 self-collected swabs for *S. aureus* culturing. Each participant received a gift card as an
142 incentive. Year 1 was excluded from this analysis as the social network index was only
143 assessed in years 2 and 3. All adult participants provided informed consent and the
144 study was approved by the Northern Arizona University Institutional Review Board.

## 145 Participants

146 Groups of two or more people who appeared to be together were invited by study
147 staff to participate in the study. Study staff confirmed that those approached were part
148 of a self-identified group. Children were eligible to participate but because their survey
149 responses were often completed by adult proxy respondents we restrict our analysis to
150 adults 18 years and over (see Figure 1). Our analytic sample size was 443 (442 for self-
151 rated health analyses) and is a subset of our previous study which included all age
152 groups (6).
153 **Fig 1.** Participant flow

## 154 Social relationship assessments

155 *Social Network Index*. The SNI (1) assesses relationships across 12 types of social
156 roles; spouse/partner, child, in-laws, parents, other close family members, friends,
157 neighbors, coworkers, school contexts, volunteer work, religious contexts, as well as a
158 broad category representing nonreligious social/recreational/professional groups. The
159 SNI also assesses the number of persons interacted with in each social role. If persons
160 interacted (by phone or in person) at least once every two weeks they received a point
161 for that social role domain and these points were summed to create the network
162 diversity score (range 0-12). Network size (the total number of social contacts across
163 those domains) was the sum of all persons with whom the respondent interacted in
164 these roles. Network size counts were naturally limited for some social roles (e.g., 1
165 person for a spouse/partner role; 2 people each for parents and in-laws) but for other
166 domains (e.g., close friends) response options ranged from zero to seven or more. The
167 number of persons in the work domain ranged up to 14 as both the number of persons
168 supervised (0-7+) and the number of nonsupervisory contacts (0-7+) were included in
169 the sum (possible range 0-75). Social network diversity, but not network size, has been
170 linked to infectious disease risk (1).
171 We modified the last SNI question related to nonreligious group membership
172 from the original (1). We added “fitness classes” as an example group activity to make
173 the examples more contemporary and made the number of contacts assessment
174 parallel to the relatives/close friends/religious group questions, i.e., capped the number
175 of contacts at 7 or more persons.
176 *Social integration and loneliness*. We used seven items from the 2001 US National
177 Health Interview Survey (NHIS) (29) that captured social contacts with persons not
178 living with the respondent. Questions referenced interactions in the past two weeks and
179 included in-person or phone contact with friends or family, participating in a group social
180 activity, attending a religious service, and eating out. The eating out variable did not
181 refer specifically to eating with others but it aligns statistically with the other social
182 integration items and including it in the index improves stratification of mortality (13). We
183 also included whether or not participants were married (which is also included in the
184 network diversity score). Questions were scored as yes/no during that interval and yes
185 responses were summed to create a social integration score. This score is strongly
186 associated with all-cause mortality (13, 14) and with psychological well-being (30).
187 Loneliness was assessed by asking “In general, how often do you feel lonely?
188 Always, usually, sometimes, never.” Similar items are used in public health surveys (31)
189 and this single item assessment is strongly associated with depression and depressive
190 symptoms even when statistically controlling for other social relationship variables (32,
191 33) and loneliness is linked to a weaker immune response to influenza vaccine (34).
192 **Detection of *S. aureus***
193 Methods and results of our sampling for *S. aureus* are detailed in Russakoff et al. (6),
194 however here, we excluded children <18 years of age. Briefly, two double tipped BBL
195 CultureSwab Swabs were used for sampling each of three different body sites (nares,
196 throat, and hand). Swabbing was supervised by study staff (who worked in pairs) who
197 also ensured that the self-swabbing was completed properly, i.e., swabbing each site for
198 twenty seconds. One double tipped swab was used for culturing while the other was
199 used for DNA-based direct detection of *S. aureus.* While in the field, all swabs were
200 stored on ice. In the laboratory, the double tipped swab destined for DNA-based
201 detection was stored at -70oC before DNA extraction and detection. For direct detection
202 of *S. aureus*, we used SaQuant, a quantitative real-time PCR assay with 95.6%
203 sensitivity and 99.9% specificity (35). The other double tipped swab, destined for
204 culturing, was stored at 4oC in the laboratory for no more than 48 hours before culturing
205 to maximize the likelihood of cell survival (36). For culturing, swabs were streaked onto
206 CHROMagar *S. aureus* media and incubated for 24 hours at 37°C. CHROMagar Staph
207 aureus chromogenic media allows for differentiation of *S. aureus* from other species as
208 *S. aureus* colonies appear pink to mauve while other bacterial species are inhibited or
209 will appear colorless or blue. Details on our efforts to ensure against false positive
210 results are detailed in Russakoff et al. (6) and include whole genome sequencing and
211 MLST v2.0 and SCC*mec*Finder v1.2 analyses (www.genomicepidemiology.org) of
212 putative *S. aureus* colonies (37). For samples that were culture negative but SaQuant
213 positive, we used an amplicon sequencing approach to verify species designation (6) as
214 well as extensive validation of the SaQuant assay to ensure accurate calls on samples
215 with low *S. aureus* quantities (35). Individuals were recorded as being colonized by *S.*
216 *aureus* if any of the swabs from any of the three body sites tested positive for *S. aureus*
217 using the SaQuant assay or culture. Culture positive samples where the paired double
218 tipped swab tested negative for SaQuant were counted as negative if the culture was
219 confirmed as not *S. aureus* by whole genome sequencing.

## 220 Data reduction and analysis

221 We collapsed categories of the social relationship variables to ensure reasonable
222 numbers of positive *S. aureus* cultures in social relationship strata and to ensure more
223 stable estimates. We created a 6 category social integration variable by combining the
224 lowest 4 groups (i.e., 0-3, 4, 5, 6, 7 & 8), a 6 category network diversity score by
225 collapsing the lowest and highest categories (0-4, 5, 6, 7, 8, 9-12) and also collapsed
226 the *often* and *always* lonely groups. Similarly, we created a 5 category network size
227 variable with approximately equal numbers in each category (cutoffs of 0-15, 16-21, 22-
228 25, 26-33, 34-65) (see Table 1). All social relationship variables met an interval
229 assumption (could be modeled as a single variable with multiple levels) (38) and were
230 therefore entered as individual terms in all regression models.
231 **Table 1**. Sample Sociodemographic Characteristics

Characteristic	Total	*S. aureus	S. aureus*

|  | | negative | positive |
| --- | --- | --- | --- |
| Participants, n | 443 | 156 | 287 |
| Age y, mean (SD) | 36.4 (15.1) | 38.7 (16.7) | 35.2 (14.0) |
| Female sex, % (N) | 52.6 (233) | 66.0 (103) | 45.3 (130) |
| Hispanic ethnicity, % (N) |  |  |  |
| No | 29.8 (132) | 20.5 (32) | 34.8 (100) |
| Yes | 68.4 (303) | 75.6 (118) | 64.5 (185) |
| Missing | 1.8 (8) | 3.8 (6) | 0.7 (2) |
| Race, % (N) |  |  |  |
| White | 66.8 (296) | 62.8 (98) | 69.0 (198) |
| Black | 1.1 (5) | 1.3 (2) | 1.0 (3) |
| Native American | 0.5 (2) | 0.6 (1) | 0.3 (1) |
| Asian | 1.6 (7) | 1.9 (3) | 1.4 (4) |
| Pacific Islander | 0.2 (1) | 0.0 (0) | 0.3 (1) |
| Other race | 4.3 (19) | 7.1 (11) | 2.8 (8) |
| Multiple race | 3.8 (17) | 2.6 (4) | 4.5 (13) |
| No preferred race | 9.9 (44) | 9.6 (15) | 10.1 (29) |
| Missing  Education level, % (N) | 11.7 (52) | 14.1 (22) | 10.5 (30) |
| < High school | 12.0 (53) | 17.9 (28) | 8.7 (25) |
| High school diploma | 14.0 (62) | 16.0 (25) | 12.9 (37) |
| Some college | 50.8 (225) | 40.4 (63) | 56.4 (162) |
| College graduate or | 23.0 (102) | 25.0 (39) | 22.0 (63) |

| higher |  |  |  |
| --- | --- | --- | --- |
| Missing | 0.2 (1) | 0.6 (1) | 0.0 (0) |
| Marital status, % (N) |  |  |  |
| Married/cohabiting | 60.7 (269) | 51.3 (80) | 65.9 (189) |
| Divorced/separated | 5.9 (26) | 7.7 (12) | 4.9 (14) |
| Widowed | 1.4 (6) | 3.2 (5) | 0.3 (1) |
| Never married | 25.3 (112) | 26.3 (41) | 24.7 (71) |
| Missing | 0.9 (4) | 1.3 (2) | 0.7 (2) |
| Employed, % (N) | 78.8 (349) | 71.2 (111) | 82.9 (238) |
| Language use at home, |  |  |  |
| % (N) |  |  |  |
| Only Spanish | 17.2 (76) | 27.6 (43) | 11.5 (33) |
| More Spanish than  English | 13.3 (59) | 15.4 (24) | 12.2 (35) |
| Equal Spanish and English | 23.3 (103) | 21.8 (34) | 24.0 (69) |
| More English than Spanish | 13.8 (61) | 14.1 (22) | 13.6 (39) |
| Only English | 32.3 (143) | 21.2 (33) | 38.3 (110) |
| Other  Language of survey, % | 0.2 (1) | 0.0 (0) | 0.3 (1) |
| (N) |  |  |  |
| Spanish | 30.0 (133) | 44.2 (69) | 22.3 (64) |

English	70.0 (310)	55.8 (87)	77.7 (223)

Social integration score,

| % (N) |  | | |
| --- | --- | --- | --- |
| 1-3 | 6.3 (28) | 9.0 (14) | 4.9 (14) |
| 4 | 6.5 (29) | 5.8 (9) | 7.0 (20) |
| 5 | 20.1 (89) | 21.2 (33) | 19.5 (56) |
| 6 | 25.3 (112) | 22.4 (35) | 26.8 (77) |
| 7 | 28.2 (125) | 30.1 (47) | 27.2 (78) |
| 8 | 13.5 (60) | 11.5 (18) | 14.6 (42) |

Social network diversity

| score, % (N) |  | | |
| --- | --- | --- | --- |
| 0-4 | 18.3 (81) | 19.9 (31) | 17.4 (50) |
| 5 | 17.8 (79) | 17.3 (27) | 18.1 (52) |
| 6 | 16.9 (75) | 18.6 (29) | 16.0 (46) |
| 7 | 17.4 (77) | 19.9 (31) | 16.0 (46) |
| 8 | 14.0 (62) | 12.2 (19) | 15.0 (43) |
| 9-12 | 15.6 (69) | 12.2 (19) | 17.4 (50) |

Social network size, %

| (N) |  | | |
| --- | --- | --- | --- |
| 0-15 | 15.1 (67) | 17.9 (28) | 13.6 (39) |
| 16-21 | 20.1 (89) | 21.8 (34) | 19.2 (55) |
| 22-25 | 16.9 (75) | 12.8 (20) | 19.2 (55) |
| 26-33 | 24.6 (109) | 27.6 (43) | 23.0 (66) |

34-65	23.3 (103)	19.9 (31)	25.1 (72)

Loneliness, % (N)

| Never | 57.1 (253) | 60.3 (94) | 55.4 (159) |
| --- | --- | --- | --- |
| Sometimes | 36.3 (161) | 31.4 (49) | 39.0 (112) |
| Often/always | 6.5 (29) | 8.3 (13) | 5.6 (16) |

Self-rated health, % (N)

| Poor/fair | 11.1 (49) | 16.0 (25) | 8.4 (24) |
| --- | --- | --- | --- |
| Good | 36.6 (162) | 35.3 (55) | 37.3 (107) |
| Very good | 36.6 (162) | 32.1 (50) | 39.0 (112) |
| Excellent | 15.6 (69) | 16.0 (25) | 15.3 (44) |
| Missing | 0.2 (1) | 0.6 (1) | 0.0 (0) |

232 Social integration score was derived from 8 items from the US National Health Interview
233 Survey. Social network diversity and network size were measured using the Social
234 Network Index (1).
235 235
236 We examined each social relationship variable individually to predict binary *S.*
237 *aureus* colonization, adjusting for age and sex. The fully adjusted models were of
238 primary interest but we also report unadjusted models. We used a Poisson regression
239 model with a log link and robust clustered variance estimates. This model estimates
240 prevalence ratios (labeled here incidence rate ratios) which are preferable to odds ratios
241 derived from logistic regression (39), particularly when the outcome is common (40).
242 The clustered variance option adjusts the statistical tests to accommodate the group-
243 level sampling. A two sided p-value of 0.05 was used to determine statistical
244 significance.

# 245 Results

246 Sociodemographic characteristics of participants are in Table 1. Most participants
247 (60.7%) were married, a slight majority (52.6%) were female, most (68.4%) reported
248 Hispanic ethnicity and about a third (30%) chose to complete the Spanish version of the
249 survey. Correlations among the social relationship indicators, self-rated health (SRH)
250 and colonization with *S. aureus* are in Table 2.
251 **Table 2**. Correlations among social relationship variables, self-rated health and
252 colonization with *S. aureus*

|  | Network  size | Social  integration | Lonely | Self-rated  health | *S. aureus*  colonized |
| --- | --- | --- | --- | --- | --- |
| Social network diversity | 0.69 | 0.53 | -0.11 | 0.17 | 0.06 |
|  | (<0.001) | (<0.001) | (0.019) | (0.001) | (0.198) |
| Social network size |  | 0.36 | -0.11 | 0.19 | 0.06 |
|  |  | (<0.001) | (0.045) | (<0.001) | (0.226) |
| Social integration |  |  | -0.19 | 0.09 | 0.05 |
|  |  |  | (<0.001) | (0.073) | (0.254) |
| Lonely |  |  |  | -0.19 | 0.02 |
|  |  |  |  | (<0.001) | (0.730) |
| Self-rated health |  |  |  |  | 0.07 |
|  |  |  |  |  | (0.159) |

253 *P*-values in parentheses. Estimates adjusted for clustering. N’s = 442-443
254 254

## 255 Social relationships and self-rated health

256 We used ordinary least squares regression with clustered variance estimates to predict
257 SRH using the 4 social relationship measures. Each of these was predictive of SRH in
258 adjusted models and all but social integration were predictive in unadjusted models (see
259 Table 3). Overall, the social relationship resources were moderately correlated with one
260 another, and as in other literature (23-25), were predictive of higher self-rated health.
261 Similar findings were observed in sensitivity analyses using an ordinal logit model (data
262 not shown).
263 **Table 3**. Coefficients regressing self-rated health on social relationship variables

Unadjusted	Adjusted for age and sex

|  | b | (95%CI) | p | b | (95%CI) | p |
| --- | --- | --- | --- | --- | --- | --- |
| Social network  diversity | 0.09 | (0.04–0.14) | 0.001 | 0.08 | (0.03–0.13) | 0.001 |
| Social network | 0.12 | (0.06–0.19) | <0.001 | 0.10 | (0.04–0.16) | 0.001 |
| size |  |  |  |  |  |  |
| Social | 0.06 | (-0.01–0.13) | 0.073 | 0.07 | (0.00–0.13) | 0.044 |

integration

Lonely	-0.28 (-0.41– -0.14) <0.001	-0.32	(-0.45– -0.19)	<0.001

264 Ordinary least squares regression adjusted for clustering. N = 442
265 CI = confidence interval
266 266
267 **Colonization with *S. aureus***
268 Overall 64.8% (*n* = 287) of the participants were colonized with *S. aureus*. Sample
269 characteristics by *S. aureus* status are in Table 1.

## 270 Social relationships and colonization with *S. aureus*

271 Neither social network diversity nor social network size were associated with *S. aureus*
272 colonization (Table 4). The NHIS social integration variable and loneliness were also
273 unrelated to *S. aureus* colonization. These patterns were consistent with and without
274 adjustment for age and sex (Table 4). Log binomial models (41) predicting *S. aureus*
275 colonization were almost identical to Poisson estimates and were also consistent when
276 using indicator social relationship variables (data not shown). Similar patterns were
277 seen when using continuous social relationship variables (Table 4) and when covarying
278 socioeconomic status markers (education and home ownership; data not shown).
279 **Table 4**. Incidence rate ratios (IRR) regressing *S. aureus* colonization on individual
280 social relationship variables

Unadjusted	Adjusted for age and sex

| IRR | | (95%CI) | p | IRR | (95%CI) | p |
| --- | --- | --- | --- | --- | --- | --- |
| Categorical  variables | |  |  |  |  |  |
| Social network 1.03  diversity | | (0.99–1.07) | 0.20 | 1.02 | (0.98–1.06) | 0.26 |
| Social network | 1.03 | (0.98–1.09) | 0.23 | 1.02 | (0.97–1.07) | 0.56 |
| size |  |  |  |  |  |  |
| Social | 1.03 | (0.98–1.08) | 0.26 | 1.03 | (0.98–1.08) | 0.24 |

integration

Lonely	1.02	(0.91–1.14)	0.73	1.02	(0.91–1.14)	0.74

Continuous

variables

| Social network  diversity | 1.03 | (0.99–1.06) | 0.12 | 1.02 | (0.99–1.06) | 0.15 |
| --- | --- | --- | --- | --- | --- | --- |
| Social network  size | 1.00 | (1.00–1.01) | 0.26 | 1.00 | (1.00–1.01) | 0.59 |
| Social  integration | 1.03 | (0.98–1.07) | 0.29 | 1.03 | (0.98–1.08) | 0.26 |

281 All estimates are adjusted for clustering. N = 443
282 CI = confidence interval
283 283

# 284 Discussion

285 We examined the association between *S. aureus* colonization and a number of social
286 relationship resources that may denote exposure to infectious agents. We found no
287 association of social network diversity with *S. aureus* colonization prevalence in a
288 community sample of naturally occurring social groups. *S. aureus* prevalence was also
289 unrelated to loneliness, social network size, and social integration. These social
290 relationship variables have previously been linked to a number of health outcomes
291 including all-cause mortality (13, 23, 42). In contrast, convergent validity was observed
292 for all social relationship indicators via their positive associations with global SRH, the
293 latter of which is both a clinical endpoint and robustly associated with mortality (20, 21,

294	43).

295 The hypothesis that more social interaction, and thus greater exposure, would be
296 associated with higher *S. aureus* prevalence, was not supported. This was a strong test
297 of the hypothesis given our sample reported a large average social network size and we
298 observed a nonsignificant and very small association of network size and *S. aureus*
299 colonization. Household-level contacts are commonly linked to colonization and
300 transmission of *S. aureus* (44-46), however little is known about the impact of social
301 relationships outside the home or healthcare settings (47). Our results support the
302 contention that social interactions outside the home (typical of social network
303 assessments) are less important than those within the home.
304 These data show that the salutary association observed between social network
305 diversity and susceptibility to cold infection (found in only one study to date) does not
306 extend to colonization with *S. aureus*. In prior work with colds, all participants were
307 intentionally exposed to one of two cold viruses (1) whereas in the present study,
308 exposure was naturally occurring. Thus, infectious challenge studies do not directly
309 evaluate the link between social ties and a the likelihood of coming in contact with an
310 infected person or b) the chance of becoming infected given that contact (2). Our study
311 did not examine clinical infection but preserved these two critical steps on the pathway.
312 Importantly, *S. aureus* colonization is outside the dominion of immune system
313 surveillance and thus is not informed by psychoneuroimmunology models.
314 Our study is unique in that we examined the above mentioned social relationship
315 resources in conjunction with *S. aureus* colonization in people living in a diverse border
316 setting in southern Arizona. Potential participants were recruited from, and completed
317 participation in public and private spaces. Enrollees completed social relationship
318 assessments and provided swabs from three body sites (hand, anterior nares, and
319 throat) for *S. aureus* detection. No other health screening or eligibility restrictions were
320 required. Thus, our data collection closely parallels the ecology from which *S. aureus*
321 infection commonly arises and adds to our understanding of community transmission
322 and prevalence by showing that social contacts as commonly assessed do not drive
323 colonization. In a larger context, our work extends prior work on the link between social
324 network resources and infection a) by examining a different infectious agent; b) by
325 incorporating a broad assessment of social relationships; and c) by sampling within a
326 multiethnic bilingual population.
327 This study has a number of strengths and limitations to consider. Colonization
328 prevalence was substantially higher in our sample (6) relative to other estimates (5).
329 This is likely because of the greater sensitivity due to sampling from 3 different body
330 sites and coupling molecular and culture-based detection methods, approaches that in
331 combination are unique to this study (6, 48). We used the same measure of network
332 diversity as in prior studies of infectious disease risk, and complemented that with
333 additional social integration assessments and a subjective social relationship measure.
334 As in other studies (49) correlations among these measures of quasi-objective and
335 subjective social relationship resources were of small to medium magnitude (except for
336 network size and diversity measures, which were strongly correlated with each other)
337 and thus provide good coverage of different social relationship domains. Our sample
338 had a similar proportion of persons across social integration categories relative to
339 representative samples (14). We had to collapse the lowest social integration categories
340 but this was expected as very low social integration is rare, observed in less than 2% of
341 the population (13).
342 Our study was cross-sectional and we did not include a clinical infectious
343 outcome. It is therefore possible that clinical *S. aureus* infection, rather than
344 colonization, is linked to social relationships. Despite this possibility, our investigation of
345 social relationship determinants of carriage in naturally occurring social groups, using a
346 rigorous assessment of *S. aureus* colonization, illuminates the extent to which social
347 relationships stratify community reservoirs of this important infectious agent.

## 348 CONCLUSIONS

349 In a sample of naturally occurring social groups within a community, we did not find a
350 statistically significant association between social network diversity and *S. aureus*
351 colonization prevalence. Our results also did not support the hypothesis that more social
352 interaction would be associated with a higher *S. aureus* prevalence. Coupled, these
353 findings suggest that, in this sample, structural social relationship characteristics and
354 loneliness are not associated with colonization of *S. aureus.* Future research on *S.*
355 *aureus* colonization should examine more intimate forms of social contact and
356 longitudinal carriage to determine sources of transmission and ultimately, targeted
357 mitigation strategies.
358 358
359 **Abbreviations**: MRSA = methicillin-resistant *Staphylococcus aureus,* MSSA =
360 methicillin-susceptible *Staphylococcus aureus,* SNI = social network index, SRH = self-
361 rated health, NHIS = National Health Interview Survey, PCR = polymerase chain
362 reaction, DNA = deoxyribonucleic acid
363 363

## 364 Competing interests

365 The authors declare that they have no competing interests.
366 366

## 367 Funding

368 This work was supported by the NAU Southwest Health Equity Research Collaborative,
369 which is funded by the National Institute on Minority Health and Health Disparities at the
370 National Institutes of Health (grant NIH U54MD012388). Funding was also received
371 from the National Institute of Allergy and Infectious Diseases at the National Institutes of
372 Health (R15AI156771) The funding body had no role in the study design, data
373 collection, analysis, interpretation or decision to submit for publication.
374 374

## 375 Authors' contributions

376 **SDB:** conceptualization, methodology, validation, formal analysis, investigation, data
377 curation, writing-original draft, funding acquisition. **MRL:** conceptualization,
378 methodology, investigation, writing-original draft, funding
379 acquisition. **RTT:** conceptualization, methodology, validation, formal analysis,
380 investigation, writing-review & editing, project administration, funding
381 acquisition. **MM:** methodology, assay validation, investigation, data curation, writing-
382 review & editing, supervision, project administration. **SK:** methodology, assay validation,
383 investigation, data curation, writing-review & editing, supervision, project
384 administration. **KT-M:** investigation, data curation, writing-review & editing draft,
385 supervision, project administration. **CW:** methodology, assay validation, investigation,
386 data curation, writing-review & editing. **BC:** assay validation, investigation, writing-
387 review & editing. **BR:** assay validation, investigation, writing-review &
388 editing. **KC:** investigation, data curation, writing-review & editing. **CP:** investigation, data
389 curation, writing-review & editing. **SM:** investigation, data curation, writing-review &
390 editing, supervision, project administration. **TP:** conceptualization, methodology,
391 validation, investigation, data curation, writing-original draft, supervision, project
392 administration, funding acquisition.
393 393

# 394 Acknowledgements

395 This work would not have been possible without the support from the Regional Center
396 for Border Health in Somerton, AZ. We are grateful to the many field surveyors from
397 NAU and the Regional Center for Border Health who recruited, sampled, and collected
398 data from participants.
399 399
400 400

# 401 References

402 1.	Cohen S, Doyle WJ, Skoner DP, Rabin BS, Gwaltney JM, Jr. Social ties and
403 susceptibility to the common cold. JAMA 1997;277:1940-4.
404 2.	Edmunds WJ, Medley GF, O'Callaghan CJ. Social ties and susceptibility to the
405 common cold. JAMA 1997;278:1231; author reply 1232.
406 3.	Tong SYC, Davis JS, Eichenberger E, Holland TL, Fowler VG. *Staphylococcus*
407 *aureus* infections: epidemiology, pathophysiology, clinical manifestations, and
408 management. Clin Microbiol Rev 2015;28:603-661.
409 4.	Kourtis AP, Hatfield K, Baggs J, Mu Y, See I, Epson E, Nadle J, Kainer MA,
410 Dumyati G, Petit S, Ray SM, Ham D, Capers C, Ewing H, Coffin N, McDonald LC,
411 Jernigan J, Cardo D. Epidemiology and recent trends in methicillin-resistant and in
412 methicillin-susceptible *Staphylococcus aureus* bloodstream infections — United States.

413	MMWR 2019;68:214-219.

414	5.	Gorwitz RJ, Kruszon-Moran D, McAllister SK, McQuillan G, McDougal LK,

415	Fosheim GE, Jensen BJ, Killgore G, Tenover FC, Kuehnert MJ. Changes in the

416	prevalence of nasal colonization with *Staphylococcus aureus* in the United States,

417	2001-2004. J Infect Dis 2008;197:1226-34.

418 6.	Russakoff B, Wood C, Lininger MR, Barger SD, Trotter RT, Maltinsky S, Mbegbu
419 M, Coyne B, Yague DP, Kyman S, Tucker-Morgan K, Ceniceros K, Padilla C, Hurtado
420 K, Menard A, Villa F, Wayment HA, Hepp C, Furstenau T, Fofanov V, Liu CM, Pearson
421 TR. A quantitative assessment of Staphylococcus aureus community carriage in Yuma,
422 Arizona. J Infect Dis 2022.
423 7.	Gualandi N, Mu Y, Bamberg WM, Dumyati G, Harrison LH, Lesher L, Nadle J,
424 Petit S, Ray SM, Schaffner W, Townes J, McDonald M, See I. Racial disparities in
425 invasive methicillin-resistant *Staphylococcus aureus* infections, 2005-2014. Clin Infect

426	Dis 2018;67:1175-1181.

427 8.	von Eiff C, Becker K, Machka K, Stammer H, Peters G. Nasal carriage as a
428 source of *Staphylococcus aureus* bacteremia. N Engl J Med 2001;344:11-16.
429 9.	Perl TM, Cullen JJ, Wenzel RP, Zimmerman MB, Pfaller MA, Sheppard D,
430 Twombley J, French PP, Herwaldt LA. Intranasal mupirocin to prevent postoperative
431 *Staphylococcus aureus* infections. N Engl J Med 2002;346:1871-7.
432 10.	Wertheim HF, Melles DC, Vos MC, van Leeuwen W, van Belkum A, Verbrugh
433 HA, Nouwen JL. The role of nasal carriage in *Staphylococcus aureus* infections. Lancet

434	Infect Dis 2005;5:751-62.

435 11.	Brissette I, Cohen S, Seeman TE. Measuring social integration and social
436 networks. In: Cohen S, Underwood L, Gottlieb B, editors. Measuring and intervening in
437 social support New York: Oxford University Press; 2000. p. 53-85.
438 12.	Berkman LF, Glass T, Brissette I, Seeman TE. From social integration to health:
439 Durkheim in the new millennium. Soc Sci Med 2000;51:843-857.
440 13.	Barger SD. Social integration, social support and mortality in the US National
441 Health Interview Survey. Psychosom Med 2013;75:510-7.
442 14.	Barger SD, Uchino BN. Racial and ethnic variation in the association of social
443 integration with mortality: ten-year prospective population-based US study. Sci Rep

444	2017;7:43874.

445	15.	de Jong Gierveld J, Havens B. Cross-national comparisons of social isolation

446	and loneliness: introduction and overview. Can J Aging 2004;23:109-13.

447	16.	de Jong-Gierveld J. Developing and testing a model of loneliness. J Pers Soc

448	Psychol 1987;53:119-28.

449	17.	Shankar A, McMunn A, Banks J, Steptoe A. Loneliness, social isolation, and

450	behavioral and biological health indicators in older adults. Health Psychol 2011;30:377-

451	385.

452	18.	DeSalvo KB, Bloser N, Reynolds K, He J, Muntner P. Mortality prediction with a

453	single general self-rated health question. A meta-analysis. J Gen Intern Med

454	2006;21:267-75.

455 19.	Goldberg P, Gueguen A, Schmaus A, Nakache JP, Goldberg M. Longitudinal
456 study of associations between perceived health status and self reported diseases in the
457 French Gazel cohort. J Epidemiol Community Health 2001;55:233-8.
458 20.	Idler EL, Benyamini Y. Self-rated health and mortality: a review of twenty-seven
459 community studies. J Health Soc Behav 1997;38:21-37.
460 21.	Barger SD, Cribbet MR, Muldoon MF. Participant-reported health status predicts
461 cardiovascular and all-cause mortality independent of established and nontraditional
462 biomarkers: evidence from a representative US sample. J Am Heart Assoc 2016;5.
463 22.	Haring R, Feng YS, Moock J, Volzke H, Dorr M, Nauck M, Wallaschofski H,
464 Kohlmann T. Self-perceived quality of life predicts mortality risk better than a multi-
465 biomarker panel, but the combination of both does best. BMC Med Res Methodol

466	2011;11:103.

467 23.	Laugesen K, Baggesen LM, Schmidt SAJ, Glymour MM, Lasgaard M, Milstein A,
468 Sorensen HT, Adler NE, Ehrenstein V. Social isolation and all-cause mortality: a
469 population-based cohort study in Denmark. Sci Rep 2018;8:4731.
470 24.	Sirven N, Debrand T. Social participation and healthy ageing: An international
471 comparison using SHARE data. Soc Sci Med 2008;67:2017-2026.
472 25.	Gorman BK, Sivaganesan A. The role of social support and integration for
473 understanding socioeconomic disparities in self-rated health and hypertension. Soc Sci

474	Med 2007;65:958-75.

475 26.	Platt JR. Strong inference: certain systematic methods of scientific thinking may
476 produce much more rapid progress than others. Science 1964;146:347-53.
477 27.	Barger SD, Lininger MR, Trotter RT, 2nd, Wayment HA, Mbegbu M, Kyman S,
478 Pearson T. Educational attainment and *Staphylococcus aureus* colonization in a
479 Hispanic border community: testing fundamental cause theory. mSphere 2020;5.
480 28.	Pearson T, Barger SD, Lininger M, Wayment H, Hepp C, Villa F, Tucker-Morgan
481 K, Kyman S, Cabrera M, Hurtado K, Menard A, Fulbright K, Wood C, Mbegbu M,
482 Zambrano Y, Fletcher A, Medina-Rodriguez S, Manone M, Aguirre A, Milner T, Trotter Ii
483 RT. Health disparities in *Staphylococcus aureus* transmission and carriage in a border
484 region of the United States based on cultural differences in social relationships: protocol
485 for a survey study. JMIR Res Protoc 2019;8:e14853.
486 29.	National Center for Health Statistics, Division of Health Interview Statistics. 2001
487 National Health Interview Survey (NHIS) public use data release: NHIS survey
488 description. Hyattsville, Maryland; 2003.
489 30.	Barger SD, Donoho CJ, Wayment HA. The relative contributions of race/ethnicity,
490 socioeconomic status, health, and social relationships to life satisfaction in the United
491 States. Qual Life Res 2009;18:179-89.
492 31.	McGinty EE, Presskreischer R, Han H, Barry CL. Psychological distress and
493 loneliness reported by US adults in 2018 and April 2020. JAMA 2020;324:93-94.
494 32.	Barger SD, Messerli-Bürgy N, Barth J. Social relationship correlates of major
495 depressive disorder and depressive symptoms in Switzerland: nationally representative
496 cross sectional study. BMC Public Health 2014;14:273.
497 33.	Sjoberg L, Ostling S, Falk H, Sundh V, Waern M, Skoog I. Secular changes in
498 the relation between social factors and depression: a study of two birth cohorts of
499 Swedish septuagenarians followed for 5 years. J Affect Disord 2013;150:245-52.
500 34.	Pressman SD, Cohen S, Miller GE, Barkin A, Rabin BS, Treanor JJ. Loneliness,
501 social network size, and immune response to influenza vaccination in college freshmen.

502	Health Psychol 2005;24:297-306.

503 35.	Wood C, Sahl J, Maltinsky S, Coyne B, Russakoff B, Panisello Yagüe D, Bowers
504 J, Talima P. SaQuant: A real-time PCR assay for quantitative assessment of
505 *Staphylococcus aureus*. Manuscript submitted for publication. 2021.
506 36.	Panisello Yague D, Mihaljevic J, Mbegbu M, Wood CV, Hepp C, Kyman S,
507 Hornstra H, Trotter R, Cope E, Pearson T. Survival of *Staphylococcus aureus* on
508 sampling swabs stored at different temperatures. J Appl Microbiol 2021.
509 37.	Pearson T, Hepp C, Trotter RT, 2nd, Mbegbu M, Russakoff B, Panisello Yague
510 D, Wood C, Tucker-Morgan K, Ceniceros K, Padilla C, Kyman S, Villa F. Genome
511 sequences of community carriage strains of Staphylococcus aureus from Yuma,
512 Arizona. Microbiol Resour Announc 2021;10:e0044921.
513 38.	Long JS, Freese J. Regression models for categorical outcomes using Stata. 2nd
514 ed. College Station, TX: Stata Press; 2003.
515 39.	Zou G. A modified poisson regression approach to prospective studies with
516 binary data. Am J Epidemiol 2004;159:702-6.
517 40.	Norton EC, Dowd BE, Maciejewski ML. Odds ratios-current best practice and

518	use. JAMA 2018;320:84-85.

519	41.	Petersen MR, Deddens JA. A comparison of two methods for estimating

520	prevalence ratios. BMC Med Res Methodol 2008;8:9.

521	42.	Holt-Lunstad J, Smith TB, Baker M, Harris T, Stephenson D. Loneliness and

522	social isolation as risk factors for mortality: a meta-analytic review. Perspect Psychol Sci

523	2015;10:227-37.

524	43.	Benyamini Y, Idler EL. Community studies reporting association between self-

525	rated health and mortality: additional studies, 1995 to 1998. Res Aging 1999;21:392-

526	401.

527	44.	Knox J, Uhlemann AC, Lowy FD. Stopping household methicillin-resistant

528	*Staphylococcus aureus* transmission and recurrent infections: an unmet challenge. Clin

529	Infect Dis 2021;73:e4578-e4580.

530 45.	Ng W, Faheem A, McGeer A, Simor AE, Gelosia A, Willey BM, Watt C,
531 Richardson DC, Wong H, Ostrowska K, Vernich L, Muller MP, Gnanasuntharam P,
532 Porter V, Katz K. Community- and healthcare-associated methicillin-resistant
533 *Staphylococcus aureus* strains: An investigation into household transmission, risk
534 factors, and environmental contamination. Infect Control Hosp Epidemiol 2017;38:61-

535	67.

536 46.	Shankar N, Chow ALP, Oon J, Hsu LY, Ang B, Pang J, De Sessions PF,
537 Periaswamy B, Tambyah PA, Teo DB, Tam CC. The epidemiology and transmission of
538 methicillin-resistant *Staphylococcus aureus* in the community in Singapore: study
539 protocol for a longitudinal household study. BMC Infect Dis 2017;17:678.
540 47.	Knox J, Uhlemann AC, Lowy FD. *Staphylococcus aureus* infections: transmission
541 within households and the community. Trends Microbiol 2015;23:437-44.
542 48.	Popovich KJ, Aroutcheva A, Hota B, Beavis KG, Hayden MK, Weinstein RA.
543 Anatomic sites of colonization with community-associated methicillin-resistant
544 Staphylococcus aureus. Infect Control Hosp Epidemiol 2014;35:1192-4.
545 49.	Coyle CE, Dugan E. Social isolation, loneliness and health among older adults. J

546	Aging Health 2012;24:1346-63.

547

Figure	[Click here to access/download;Figure;Fig1.tiff](https://www.editorialmanager.com/pone/download.aspx?id=32447624&guid=48f26a57-607b-42c9-9166-f447b2ae0f01&scheme=1)
